# Global mental health and development: a thematic series

**DOI:** 10.1186/1752-4458-8-27

**Published:** 2014-07-07

**Authors:** Harry Minas

**Affiliations:** 1 Global and Cultural Mental Health Unit, Centre for Mental Health, Melbourne School of Population and Global Health, The University of Melbourne, Parkville, Victoria 3010, Australia

## 

With publication of the paper “A Position Statement on mental health in the post-2015 development agenda”, [1] we launch the first International Journal of Mental Health Systems thematic series, *Global Mental Health and Development*.

The United Nations Millennium Declaration, [2] adopted by the UN General Assembly in September 2000, set out “certain fundamental values” – freedom, equality, solidarity, tolerance, respect for nature, and shared responsibility – considered essential to international relations in the twenty-first century, and key objectives of “special significance”. These included commitments to peace, security and disarmament; development and poverty eradication; protection of the environment; human rights, democracy and good governance; protecting the vulnerable; meeting the special needs of Africa; and strengthening the United Nations. In many of these areas the world has made little if any progress.

The standout success among these ambitious objectives is the domain of development and poverty eradication. The adoption by governments and development institutions of the eight Millennium Development Goals (MDGs) has been followed by unprecedented effort and investment and has resulted in impressive gains in several of the MDGs [3]. Reduction of extreme poverty and the number of people living in urban slums, major advances in HIV-AIDS, malaria and tuberculosis, improvements in maternal health, and substantial increases in the number of children receiving primary education are examples of development gains that have a direct and positive impact on population mental health [4].

However, it is also clear that some of the most vulnerable and marginalised populations, including people with mental disorders and psychosocial disabilities (and people with physical, sensory, or intellectual disabilities), have been largely excluded from the benefits of these broad gains. “If one goes into the poorest urban slum or the most marginalized rural village and asks ‘who is the poorest person in your community?’ one will almost invariably be directed to the household of a person with a disability [5].”

Now, with 2015 and the end of the MDGs program looming, there is a renewed sense of urgency as countries race towards the finish line. For the past several years the UN has been working with governments and a vast array of stakeholders on MDG Acceleration [6] and on framing the development agenda beyond 2015. The broad structure of the post-2015 development agenda has been outlined in *A New Global Partnership*, the report of the High-Level Panel of Eminent Persons on the Post-2015 Development Agenda [7]. There are several aspects of this agenda that are important for global mental health. For example, the commitment to “leave no-one behind… and to achieve a pattern of development where dignity and human rights become a reality for all, where an agenda can be built around human security” [8]. This commitment comes from recognition that some of the most vulnerable groups have been virtually invisible and have not benefited from the current approach to development. The intention to “tackle the causes of poverty, exclusion and inequality” [7] cannot be achieved without effective attention to the poverty, exclusion and inequality that is the everyday experience of people with mental disorder and disability, and the social attitudes and political, economic and institutional arrangements that give rise to such misery.

Progress has been made in global mental health [9]. We can now reasonably claim to know what needs to be done to improve the mental health of populations but, in most parts of the world we are still a long way from implementing what we know. The reasons for implementation failure are not hard to find. At the centre of most of these reasons is a failure to accord sufficient political priority to population mental health [10] and to investment in the development of mental health systems that include attention to mental health promotion, illness prevention and the delivery of treatment and disability services that are effective, accessible, affordable and culturally appropriate.

It is also the case that where there are major deficiencies in mental health there are usually also major deficiencies in general health and social support and protection systems. Effective mental health systems cannot be built in isolation from general health systems, or in isolation from social support and protection systems that ensure protection from extreme poverty and that ensure access to safe housing, dignified employment, education and training opportunities, and protection of human rights [11].

While there is increasingly widespread political support for mental health the investment necessary for development of comprehensive mental health systems has been slow in coming. The primary source of such investment must, of course, be national governments, although civil society organisations and the private sector also have vitally important roles to play. Success in the drive for universal health coverage [12] will be particularly important for people with mental disorders. For those countries that cannot independently fund such development from state resources and are reliant on donor funds the only funding that will be of sufficient scale, and that can be seamlessly connected to other related areas of development, will be from development banks, multilateral and bilateral development agencies and major philanthropic foundations. Building effective mental health systems must be linked to building effective general health and social systems and to national development programs.

The Movement for Global Mental Health (MGMH) describes its core aim as being to “improve services for people living with mental health problems and psychosocial disabilities worldwide, especially in low- and middle-income countries where effective services are often scarce. Two principles are fundamental to the Movement: scientific evidence and human rights.” These are ambitious goals. Although some see these goals as overly narrow and medicalised [13] or culturally imperialist [14] this is a gross and puzzling misreading of the intent and work of people and organisations affiliated with MGMH. It is clear to all, and certainly to MGMH, that improving services for people with mental health problems and psychosocial disabilities, and protecting human rights, will require progress in a large number of areas, many of which are outside, and appropriately beyond the control, of health systems. It is very clear that the kinds of coherent political, economic and social (including cultural) actions that are envisaged as part of the post-2015 development agenda [7] and of the MGMH position statement [1] are essential for progress in global mental health [4].

We now invite submission of manuscripts - research articles and reviews, perspective and debate articles, case studies, guidelines and research protocols - that will illuminate how the core concerns and objectives of global mental health can be effectively integrated with those of the post-2015 development agenda. We will be particularly interested to receive manuscripts focusing on work that seeks to implement the recommendations of the MGMH position statement, [1] the WHO Action Plan [15] and the Convention on the Rights of Persons with Disabilities [11].

The MGMH Position Statement asserts that attention to mental health is essential for the success of the post-2015 development agenda. The issues raised and the recommendations made in the statement are deserving of detailed, critical and creative exploration.

## Author information

Head, Global and Cultural Mental Health Unit, Centre for Mental Health, Melbourne School of Population and Global Health, The University of Melbourne; and Editor-in-Chief, International Journal of Mental Health Systems.
